# Research on aerodynamic performance of centrifugal compressors for hydrogen-mixed natural gas

**DOI:** 10.1371/journal.pone.0312829

**Published:** 2024-10-28

**Authors:** Jingya Dong, Bin Song, Xiaoyun Yuan, Wanchun Jin, Jin Wang

**Affiliations:** 1 Chinese Petroleum Corporation Natural Gas Engineering Research Institute of Southwest Oil and Gas Field Company, Chengdu, China; 2 Key Laboratory of Natural gas Quality Control and Energy measurement for state market regulation, State Administration for Market Regulation, Chengdu, China; 3 School of Mechatronic Engineering, Southwest Petroleum University, Chengdu, China; Henan Polytechnic University, CHINA

## Abstract

Natural gas-doped hydrogen transportation has been widely used in industrial engineering. The change of the physical parameters of the conveying medium affects the working performance of the centrifugal compressor. This study aims to explore the aerodynamic performance of centrifugal compressors for hydrogen mixed natural gas, including the effects of hydrogen blending ratio (HBR) and inlet temperature on the pressure ratio and isentropic efficiency of compressors. The numerical simulation method is used and the results are compared with the experimental results to determine the reliability of the numerical simulation method. The results show that the pressure ratio decreases with the increase of HBR where the inlet temperature and rotation speed are constant. The pressure ratio and efficiency of the compressor are the highest when the conveying medium is pure natural gas (HBR = 0). The maximum pressure ratio reduction is 4.8% when the HBR is 5% and the inlet temperature is increased by 20 K. In summary, when the conveying medium of the compressor changes from pure natural gas to hydrogen-mixed natural gas, the working range and efficiency of the compressor will be reduced. Therefore, it is necessary to increase the rotation speed of the compressor or redesign the centrifugal compressor for hydrogen-mixed natural gas in order to achieve a constant flow rate at the outlet of the compressor.

## 1. Introduction

With the depletion of fossil energy and climate deterioration, green energy production technology which solves the problem of fossil energy depletion and emissions is developing rapidly [1–3]. As one of the most potential green energy sources, hydrogen energy is called ‘ultimate energy’ because of its abundant raw materials, high energy density and pollution-free emission [4]. Therefore, hydrogen production technology from biomass gasification has been widely concerned [5–7]. There are mainly thermochemical conversion method and microbial method for hydrogen production from biomass gasification, both of which have the advantages of low cost and high production [8]. Meanwhile, the maturity of thermochemical conversion technologies, such as electrolytic water hydrogen production and solar water splitting hydrogen production, have also greatly promoted the development of hydrogen energy. However, there are some disadvantages in the practical application of hydrogen, such as mismatch of production and marketing sites, less long-distance transportation infrastructure, high transportation risk and high transportation cost [9]. In contrast, the technology of natural gas mixed with hydrogen not only solves the problem of transportation cost of hydrogen energy, but also solves the problem of natural gas resource depletion to a certain extent. Furthermore, hydrogen-mixed natural gas can reduce carbon emissions during natural gas combustion, which is in line with the world energy development strategy [10–12]. With the development of hydrogen-mixed natural gas technology, the changes in physical properties and flow characteristics caused by hydrogen-mixed natural gas have become a hot topic which studied widely by worldwide scholars and engineers.

Theoretical researches and practical applications have shown that the physical properties of natural gas after mixed hydrogen have great changes. Yan et al. [13] simulated the mixing process of four kinds of natural gas and hydrogen. The results presented that the higher the proportion of the heavy hydrocarbon in natural gas, the longer the distance required for the uniform mixing of natural gas and hydrogen in the pipeline. Li et al. [14] studied the Joule-Thomson (J-T) coefficient of natural gas under six different hydrogen blend ratios (HBRs) and thermodynamic conditions. The results show that the J-T coefficient of hydrogen-mixed natural gas shows approximate linear decline with the increase of hydrogen blend ratio. When the hydrogen blend ratios reaches 30%, the J-T coefficient of the mixture is 40–50% lower than that of pure natural gas. Mahdi et al. [15] studied the physical properties of different components of natural gas mixed with hydrogen. It is found that with the increase of hydrogen blend ratios, the relative density, high and low calorific values of the hydrogen-mixed natural gas all decrease. Nonetheless, the upper and lower flammability limits of the mixed gas both increase. In addition, the results indicate that the energy consumption of compressors in booster stations for hydrogen-mixed natural gas is more than that of pure natural gas when providing the same calorific value transportation. It is necessary to analyze aerodynamic performance of centrifugal compressors for hydrogen mixed natural gas in order to explore the effect mechanism of hydrogen mixing.

Previous studies have shown that numerical simulation is one of the popular methods to investigate the performance of centrifugal compressors due to its high efficiency [16, 17]. Elias et al. [18] used the large eddy simulation (LES) method to simulate the centrifugal compressor and discussed generation mechanisms of its rotating stall and surge. Natural convection and thermal radiation are neglected by Gu et al., [19] and they used SST turbulence model to study the influence of external and internal heat transfer on the performance of centrifugal compressor. The reliability of the numerical method was verified by comparison with experimental data. Guo et al. [20] used the *k-ε* turbulence model to simulate the stall flow in centrifugal compressor with vaneless diffuser. The performance curves obtained by steady and transient calculation were compared with the experimental results, it’s shown that the numerical results were basically consistent with the experimental results. With the development of hydrogen mixing technology, there are more and more researches on compressive behaviors and mechanisms of hydrogen-mixed natural gas nowadays. The performance prediction model and dynamic simulation model of hydrogen-mixed natural gas pipelines were established by Peng et al. [21], and the effect of hydrogen mixing on the performance of centrifugal compressor was carried out. The results have been shown that the hydrogen-mixed natural gas could cause the performance curve of the centrifugal compressor to move down.

In summary, it is common to use CFD software to study the effect of hydrogen blending ratio on the performance of natural gas pipeline network, but the internal fluid flow and evolution mechanism of compressors after hydrogen mixed are rarely reported in the literature. At the same time, there is little literature on improving the design pressure ratio and efficiency of compressors when the transport medium becomes hydrogen-doped natural gas. In order to better understand the effects of hydrogen mixing on fluid flow and evolution mechanism of compressors, the aerodynamic model of centrifugal compressor for hydrogen-mixed natural gas is established and solved numerically based on the k-ε turbulence model in present study. The effects of hydrogen blending ratio and inlet temperature on compressor pressure ratio and efficiency were studied in detail. It provides theoretical support for the design of compressors whose medium is hydrogen-doped natural gas.

## 2. Model establishment and validation

### 2.1. Physical model

**Fig 1:**
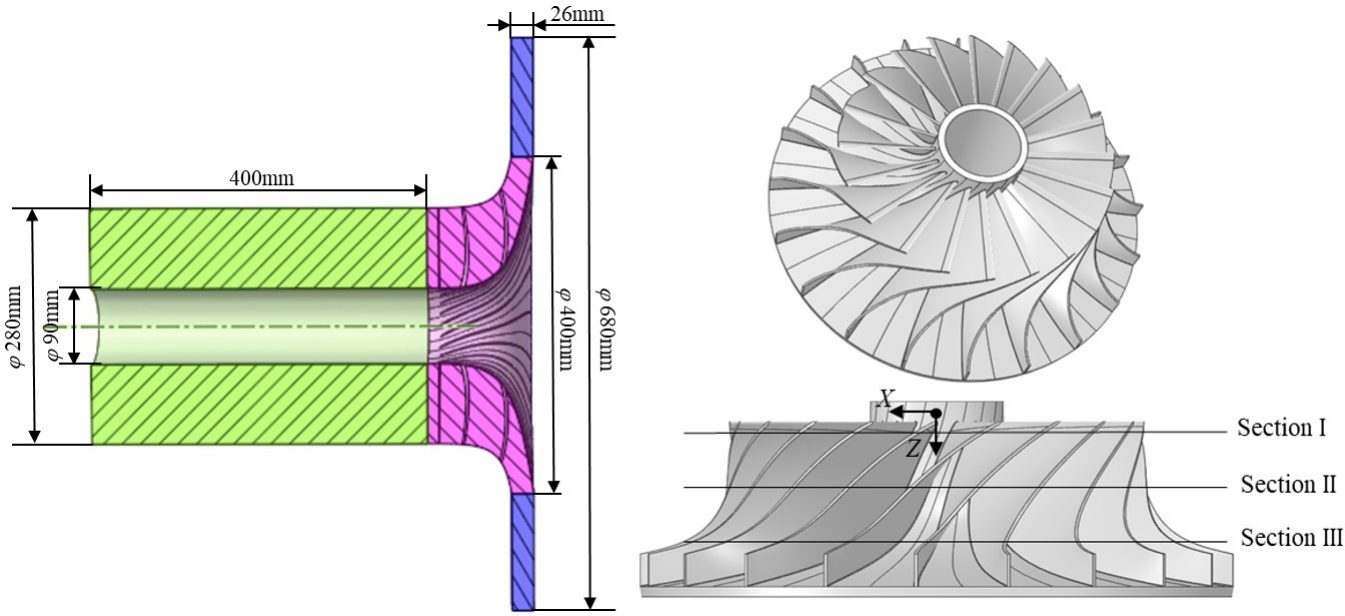
3D model of the compressor.

Eckardt [22, 23] experimentally investigated the aerodynamic performance of centrifugal compressor with specific dimensions of the impeller meridian plane and the vaneless diffuser. In order to verify the effectiveness of present numerical model, centrifugal compressor with the Eckardt’s impeller was chosen as shown in Fig 1. It can be seen that the compressor inlet fluid domain is extended by 400 mm in order to the stability of the compressor inlet flow. The impeller outlet diameter is 400 mm with its blade angle is 90°. The number of blades is 20, the tip clearance is 0.525 mm, the rated speed is 14000 rpm, and the outlet outer diameter of the compressor is 680 mm. The coordinate origin is set near the impeller inlet, and the axial distances Z corresponding to sections I, II, and III are 16 mm, 53 mm, and 90 mm.

### 2.2. Governing equations and boundary conditions

The establishment of the governing equations is the basis for solving the centrifugal compressor. The flow medium inside the compressor is a compressible fluid of hydrogen-mixed natural gas. Therefore, the following assumptions are established:

1. The gas medium flow is a continuous steady flow based on the ideal gas model.
2. The influence of gravity isn’t taken into account.
3. The influence of radiation heat transfer is ignored.

Based on the above assumptions, the governing equations are as follows: continuity equation based on the law of mass conservation is given by

$$\frac{\partial }{\partial x_{i}}\left(\rho u_{i}\right)=0$$ (1)

where *ρ* is the gas density, *u*_*i*_ is the vector of velocity.

The momentum equation without considering gravity [24] is obtained by

$$\frac{\partial }{\partial x_{j}}\left(\rho u_{i}u_{j}\right)=-\frac{\partial p}{\partial x_{i}}+\frac{\partial }{\partial x_{j}}\left[\mu \left(\frac{\partial u_{i}}{\partial x_{j}}+\frac{\partial u_{j}}{\partial x_{i}}-\frac{2}{3}\delta_{ij}\frac{\partial u_{j}}{\partial x_{i}}\right)\right]$$ (2)

where *i* and *j* are the direction vectors of the parameters. *u*_*i*_ and *u*_*j*_ are the components of fluid velocity. *p* is pressure and *δ*_*ij*_ is a unit tensor. When *i* = *j*, *δ*_*ij*_ = 1.0, When *i* ≠ *j*, *δ*_*ij*_ = 0. *μ* is the dynamic viscosity of the fluid.

Energy equation based on the law of energy conservation is calculated as

$$\frac{\partial }{\partial x_{i}}\left(\rho C_{p}u_{i}T\right)=\frac{\partial }{\partial x_{i}}\left(\left(\lambda +\lambda_{t}\right)\frac{\partial T}{\partial x_{i}}\right)$$ (3)

where *C*_*p*_ is the specific heat capacity and *T* is the temperature. *λ* and *λ*_*t*_ are thermal conductivity and turbulent thermal conductivity, respectively.

The gas state equation is used in the calculation of compressible fluid of the ideal gas model as

ρ=p/RT (4)

where *R* is the molar gas constant and *T* is the absolute temperature.

The hydrogen-mixed natural gas involves the fluid flow of multi-species mixed gas, so the steady-state species transport equation [25] is also chosen as following

$$\nabla \cdot \left(\rho u_{i}Y_{i}\right)=-\nabla \cdot J_{i}+R_{i}+S_{i}$$ (5)

where *Y*_*i*_ is mass fraction of the *i*^th^ species. *J*_*i*_ is the diffusion flux of species, which arises due to gradients of concentration and temperature. *R*_*i*_ is the net rate of production of species by chemical reaction (described later in this section) and *S*_*i*_ is the rate of creation by addition from the dispersed phase plus any user-defined sources.

Furthermore, the boundary condition of the inlet is the pressure inlet, and the outlet is the mass flow outlet. The Coupled algorithm is selected for the pressure-velocity coupling solver, and the second-order upwind scheme is used for the numerical solution of momentum, pressure and energy equations. The *k-ε* turbulence model has advantages in calculating the aerodynamic performance of the compressor [26]. All cases in this study are calculated using the *k-ε* turbulence model. The rotation domain speed is set to 14000 revolutions per minute (rpm) using the multi-reference method (MRF).

### 2.3. Model verification

#### 2.3.1. Mesh division and mesh independence verification

**Fig 2:**
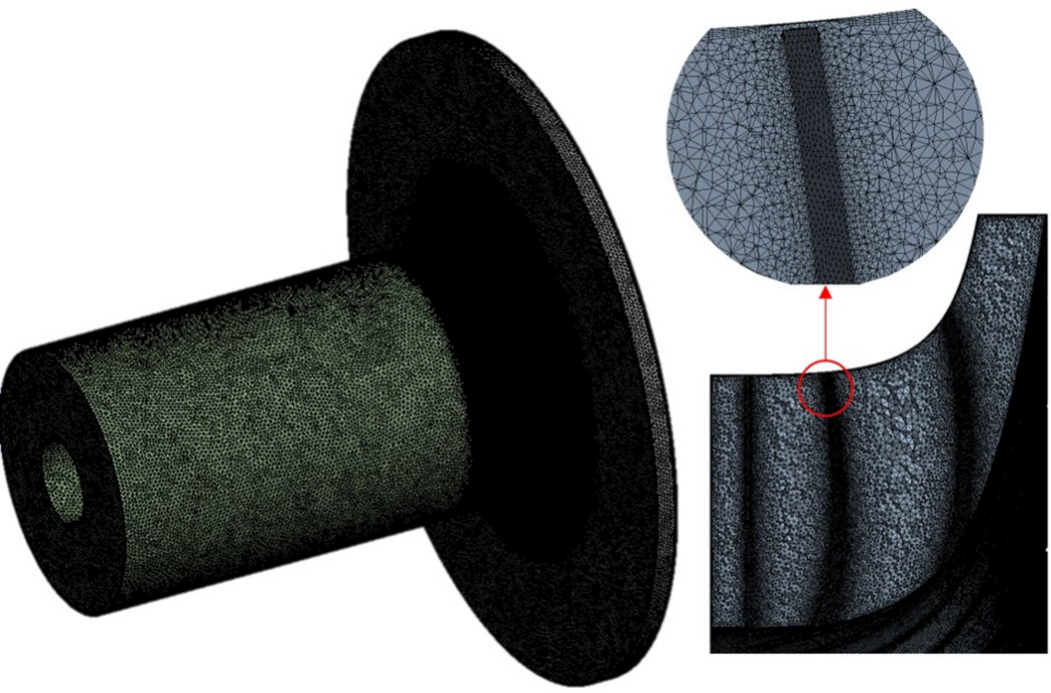
Mesh division.

Fig 2 shows the mesh division of tetrahedrons of the three-dimensional calculation model. It can be seen that the boundary layer grid is added at the impeller wall according to the Y^+^ < 300. Considering that the tip clearance is very small at 0.525 mm, the tip clearance and the impeller fluid domain are locally densified to ensure the accuracy of the compressor aerodynamic simulation.

In order to eliminate the influence of the mesh number on the calculation results, three different mesh numbers are divided for the physical model. The numerical calculation is carried out when the compressor flow rate is 5.31 kg/s, the speed is 14000 rpm, and the medium is air. The calculation results are given in Table 1. It can be found that the maximum relative error of the pressure ratio is 3.38%, and the maximum relative error of the outlet temperature is 0.6%. Therefore, the grid has little effect on the calculation results. In order to avoid the waste of computer resources, the number of 25.41 million grids is enough to capture the distribution of the physical field inside the compressor. Therefore, the number of grids in the subsequent calculation conditions of this study is 25.41 million.

**Table 1:** Mesh independence verification.

Grid Number (million)	Pressure ratio	Outlet temperature (K)
7.76	1.93	359.7
25.41	1.95	357.2
37.55	2.05	356.5
46.08	2.06	356.7
Maximum relative error	3.38%	0.6%

#### 2.3.2. Numerical verification

**Fig 3:**
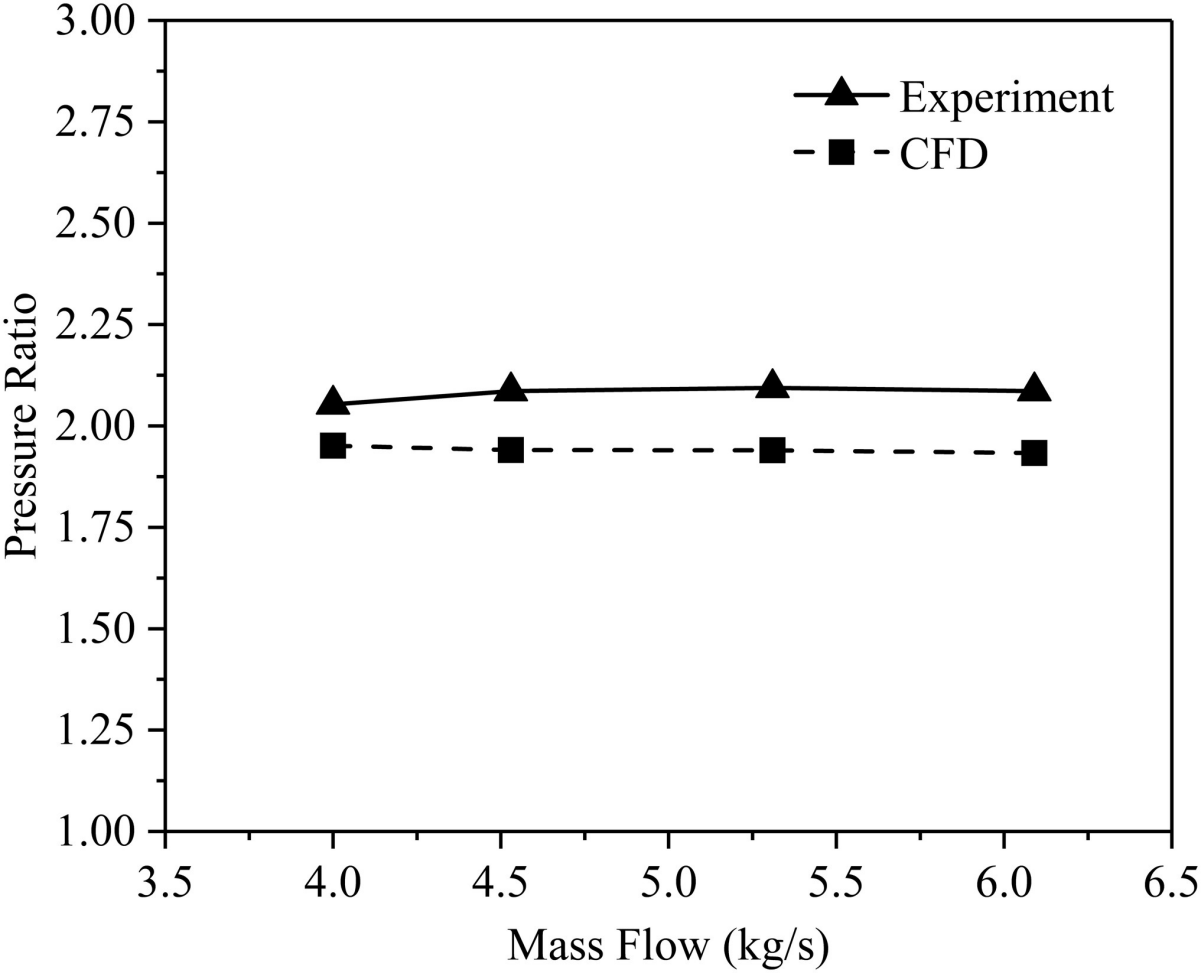
Numerical verification.

In order to verify the accuracy of the numerical calculation results, the flow rate and pressure ratio of the compressor are calculated at the rated speed of 14000 rpm. The calculated results are compared with the experimental data measured by Eckardt [22, 23]. Fig 3 shows the comparison results of present study (CFD) and the Eckardt experiment. It can be found that the distribution trend of the pressure ratio calculated by simulation and experiment is basically the same, and the simulation error is less than 8%. In addition, when the mass flow rate is 4.53kg/s, 5.31kg/s and 6.09kg/s, the simulation values of the isentropic efficiency of the compressor are 82.6%, 83.2% and 81.8%, respectively. Compared with the experimental values of 86.5%, 88% and 86.8%, it is found that the maximum error of isentropic efficiency is 5.73%. The factors that cause the above errors include model simplification, grid error, turbulence model, algorithm and so on. However, the maximum error is within the acceptable range. This numerical calculation method for the established model has certain reliability.

## 3. Factors affecting the aerodynamic performance of compressors

There are many factors that affect the aerodynamic performance of centrifugal compressors for hydrogen-mixed natural gas. This study focuses on the influence of HBR and inlet temperature on the aerodynamic performance of compressors when the conveying medium is pure natural gas and natural gas mixed with hydrogen.

The main component of natural gas is methane. The proportion of other components of methane is much lower than that of methane, so it is assumed that there is only methane for natural gas. Different mass fractions of hydrogen were mixed with natural gas to study the effect of HBR on the aerodynamic performance of centrifugal compressor. Physical properties of hydrogen and natural gas are referred to NIST REFPROP which is a software of physical property query with a very high accuracy [27]. Table 2 shows the physical parameters of the mixture with different HBR when the temperature is 288.15K, the pressure is 101325Pa. It should be noted that the density of the hydrogen-mixed natural gas in the actual simulation calculation is given according to the ideal gas state equation.

**Table 2:** Properties of hydrogen and methane mixed at different mass fractions.

Mass Fraction		Density kg/m^3^	Cp J/(kg∙K)	Thermal Conductivity W/(m∙K)	Viscosity Pa∙s	Specific	Molar
Hydrogen	Methane					Heats	Ratio (H_2_:CH_4_)
0	1	0.671	2210.00	0.03259	1.08E-05	1.3104	/
0.05	0.95	0.49718	2811.60	0.058356	1.06E-05	1.3332	0.4211
0.1	0.9	0.39498	3413.90	0.078	1.05E-05	1.3486	0.89

### 3.1. Hydrogen Blending Ratios (HBRs)

**Fig 4:**
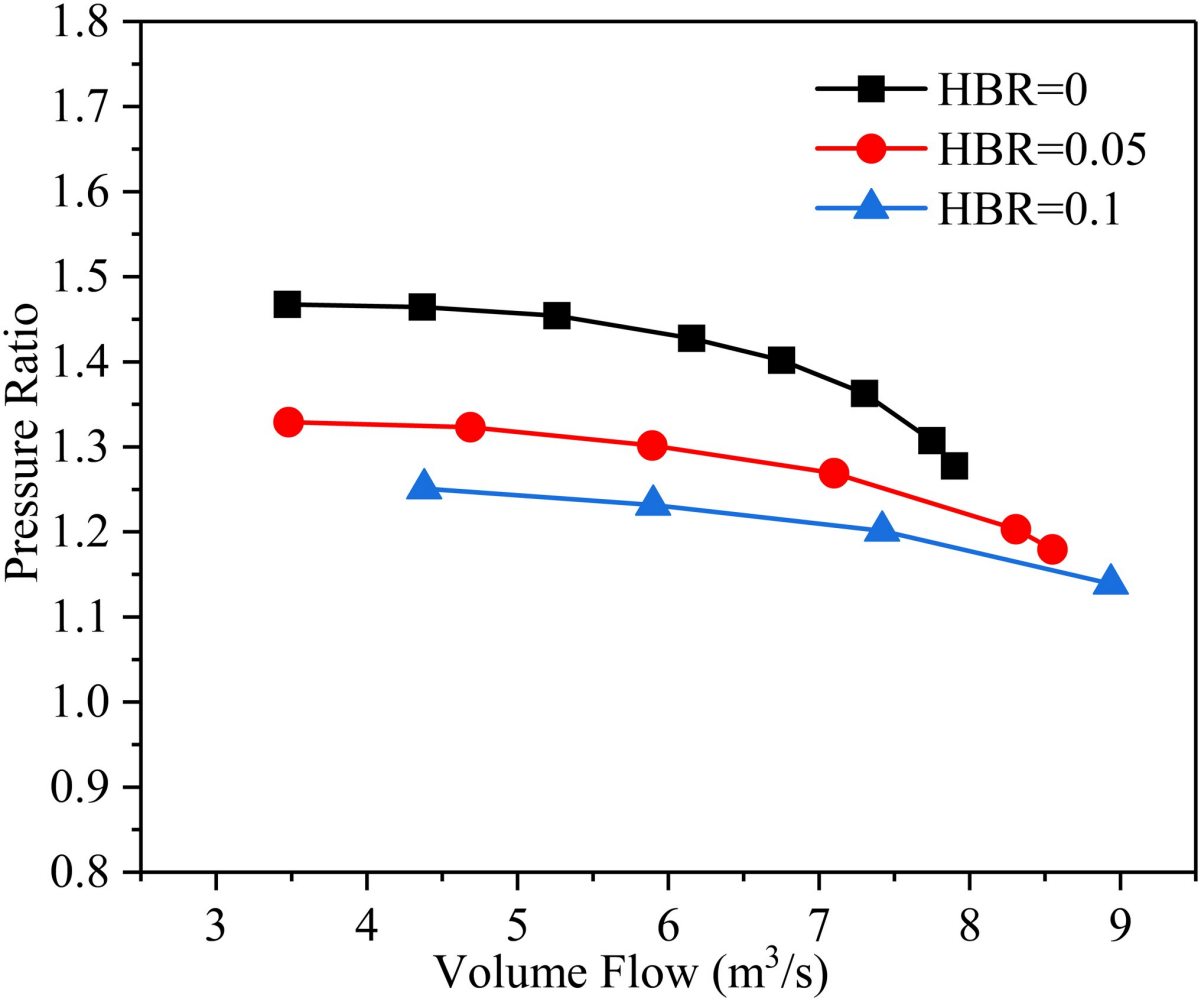
The relationship between pressure ratio and volume flow rate corresponding to different HBRs.

The relationship between the pressure ratio and the volume flow rate corresponding to different HBRs is shown in Fig 4, where the inlet temperature is 288.15 K and the impeller rotation speed is 14000 rpm. It is shown that the pressure ratio decreases with the increase of volume flow rate. When the inlet temperature and rotational speed are constant, it means that the pressure ratio is the largest when the flow medium is pure natural gas (HBR = 0) and the working range of the compressor is wider. As the HBR increases, the pressure ratio gradually decreases and the working range of the compressor also decreases. It indicates that the aerodynamic characteristics of the compressor decrease when the conveying medium changes from pure natural gas to hydrogen-mixed natural gas. Therefore, it could increase the rotational speed in order to ensure that the outlet flow of the compressor for hydrogen-mixed natural gas is consistent with that of pure natural gas.

To investigate the working efficiency of the compressor, the isentropic adiabatic efficiency is defined as:

$$\eta =\frac{T_{\text{1}}\left[{\left(\varepsilon \right)}^{\frac{\kappa -1}{\kappa }}-1\right]}{\Delta T}$$ (6)

Where Δ*T* = *T*_2_ − *T*_1_, *T*_1_ and *T*_2_ are the inlet and outlet temperature of the compressor. *ε* is pressure ratio, *κ* is the gas adiabatic index.

**Fig 5:**
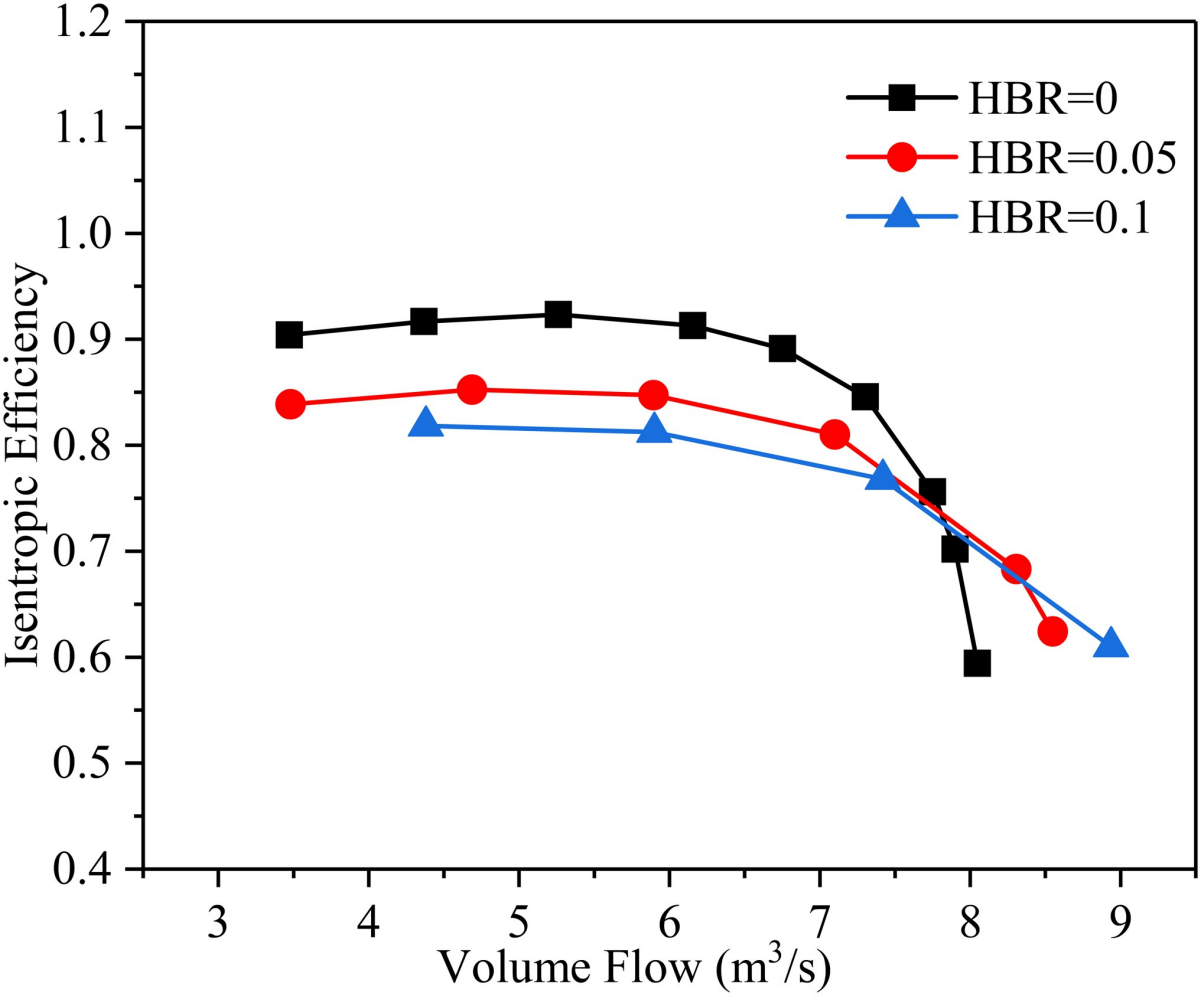
The relationship between isentropic efficiency and volume flow rate corresponding to different HBRs.

Fig 5 is the relationship between isentropic efficiency and volume flow rate corresponding to different HBRs, where the inlet temperature is 288.15 K and the impeller speed is 14000 rpm. It is found from Fig 5 that the isentropic adiabatic efficiency decreases with increasing HBR. The larger the HBR, the faster the efficiency decreases. This is because the specific heat of the mixed gas decreases with the increase of the HBR, and the surge margin and pressure ratio of the compressor also decrease. Increasing the HBR can shorten the stable working range of the compressor to a certain extent and weaken the compression ability of the gas. When the volume flow rate increases, the compressor may work outside the choke line so that the isentropic adiabatic efficiency is significantly reduced. The compressor efficiency reaches the highest value of 92.3% when the working medium is pure natural gas (HBR = 0) and the volume flow rate is 5.26 m^3^/s.

**Fig 6:**
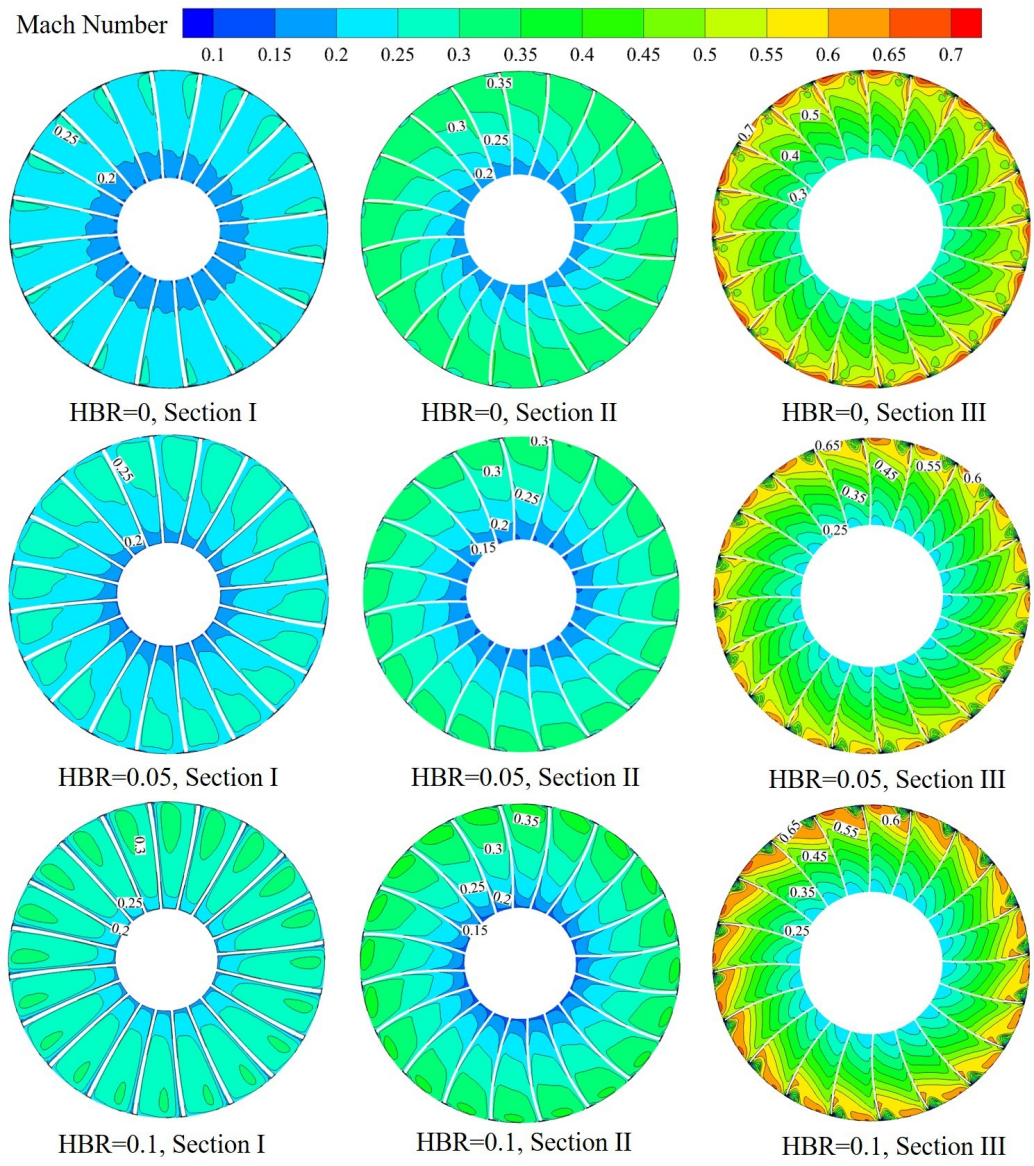
The Mach number cloud distribution on three cross sections at different HBRs.

The flow between the blades during the rotation of the impeller is a complex turbulent flow. In order to study the velocity distribution of hydrogen-mixed natural gas between blades, Mach number is used to investigate the flow behaviors inside the impeller. Fig 6 presents the distribution of Mach number on the sections I, II and III corresponding to different HBRs, where the mass flow rate is 2.93 kg/s. It can be seen that under the same HBR, the distribution of Mach number increases gradually along the Z-axis direction. For the same cross section, the distribution of Mach number varies with different HBRs. The maximum value of Mach number for section I increases with the increase of HBR. The maximum value of Mach number for section III decreases with the increase of HBR. This is due to the reduction of the pressure ratio of the compressor after hydrogen blending. The velocity of the medium flow is lower than that without hydrogen, and the Mach number is also reduced. The high Mach number is concentrated between the tip clearances, and the hydrogen-doped natural gas with high viscosity makes the blade channel blocked seriously, which makes the compressor performance decrease.

Tip-speed Mach number is an important parameter in compressor similarity design. In both a polytropic analysis and an isentropic analysis, the pressure ratio is determined by the tip-speed Mach number, the efficiency and the work coefficient. The pressure ratio is defined as [28]:

$$\pi_{t21}={\left[1+\left(\gamma -1\right)\lambda M_{u2}^{2}\right]}^{n/n-1}$$ (7)

where *γ* is specific heats and *γ* = *c*_*p*_/*c*_*v*_. *λ* is work coefficient and the typical range of radial impeller work coefficient is 0.6~0.8. *M*_*u*2_ is tip-speed Mach number and $M_{u2}=u_{2}/\sqrt{\gamma RT_{1}}$. *n* is polytropic exponent and *n*/(*n* − 1) = (*η*_*p*_*γ*)/(*γ* − 1). *η*_p_ is polytropic efficiency, it is defined as:

$$\eta_{p}=\frac{\kappa -1}{\kappa }\frac{\text{ln}\left(p_{2}/p_{1}\right)}{\text{ln}\left(T_{2}/T_{1}\right)}$$ (8)

where *κ* is isentropic exponent. The isentropic exponent, *κ*, can be replaced by the specific heats, *γ*, when using the ideal gas model.

**Fig 7:**
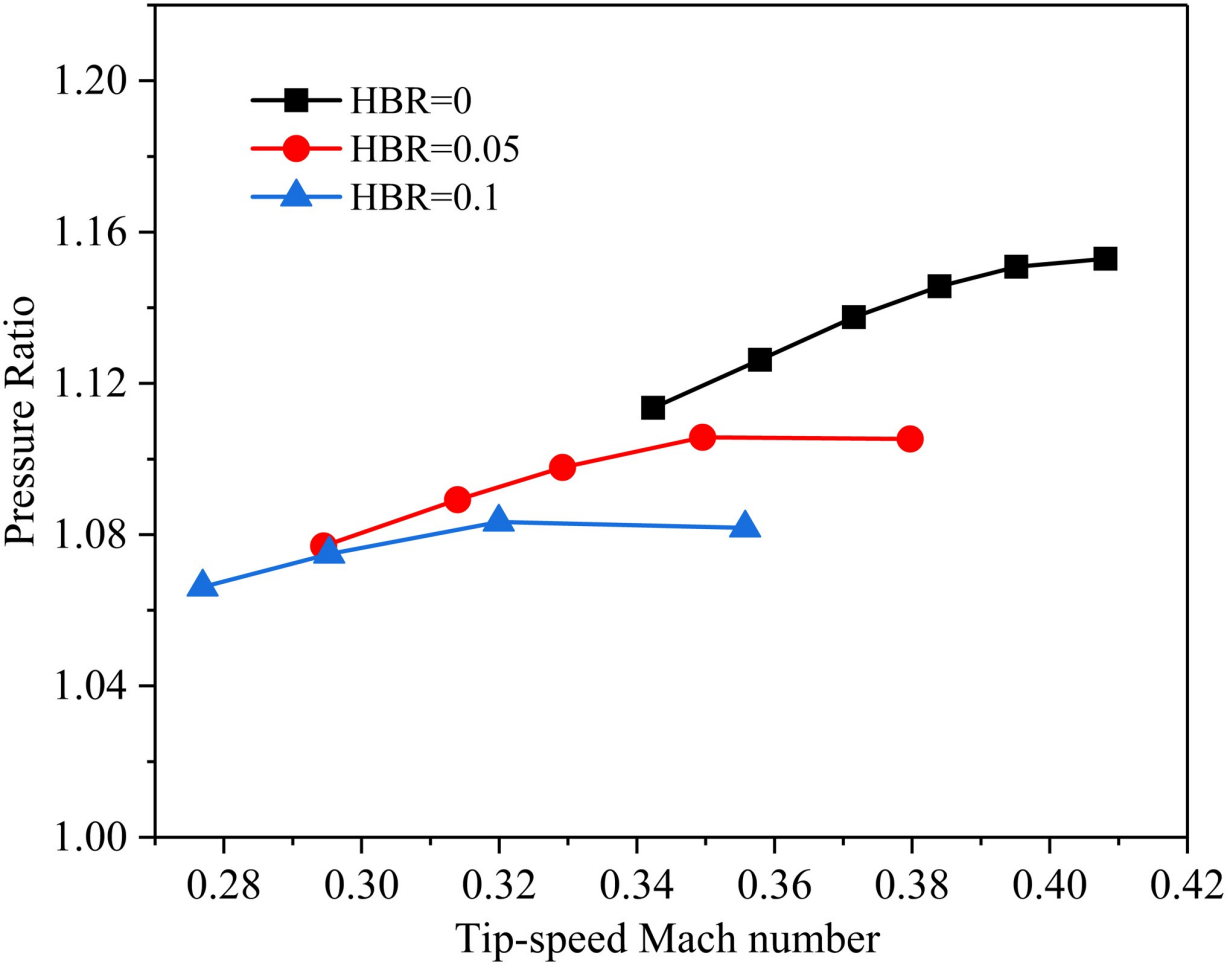
The pressure ratio is distributed with the tip-speed Mach number at different HBRs.

The rotation speed of the impeller is 14000rpm. The relationship between the tip-speed Mach number and the pressure ratio is shown in Fig 7. It can be found that the pressure ratio gradually increases with the increase of tip-speed Mach number, no matter what kind of working medium. However, the pressure ratio decreases with the increase of hydrogen doping ratio. When different working fluids have the same pressure ratio, the higher the HBR, the larger the corresponding tip-speed Mach number. This is because as the HBR in natural gas increases, the molecular weight of the working fluid decreases, which increases the speed of the sound in natural gas.

The compressor reduced mass flow rate is defined as:

$$m_{\text{red}}=m\sqrt{\frac{T_{\text{1}}}{T_{\text{ref}}}}\frac{p_{ref}}{p_{1}}$$ (9)

where *m* is compressor mass flow rate. The *T*_1_ is the inlet temperature and *p*_1_ is the inlet total pressure. The reference temperature (288.15K) and pressure (101325Pa) are often taken as those at the international standard atmospheric conditions at sea level.

**Fig 8:**
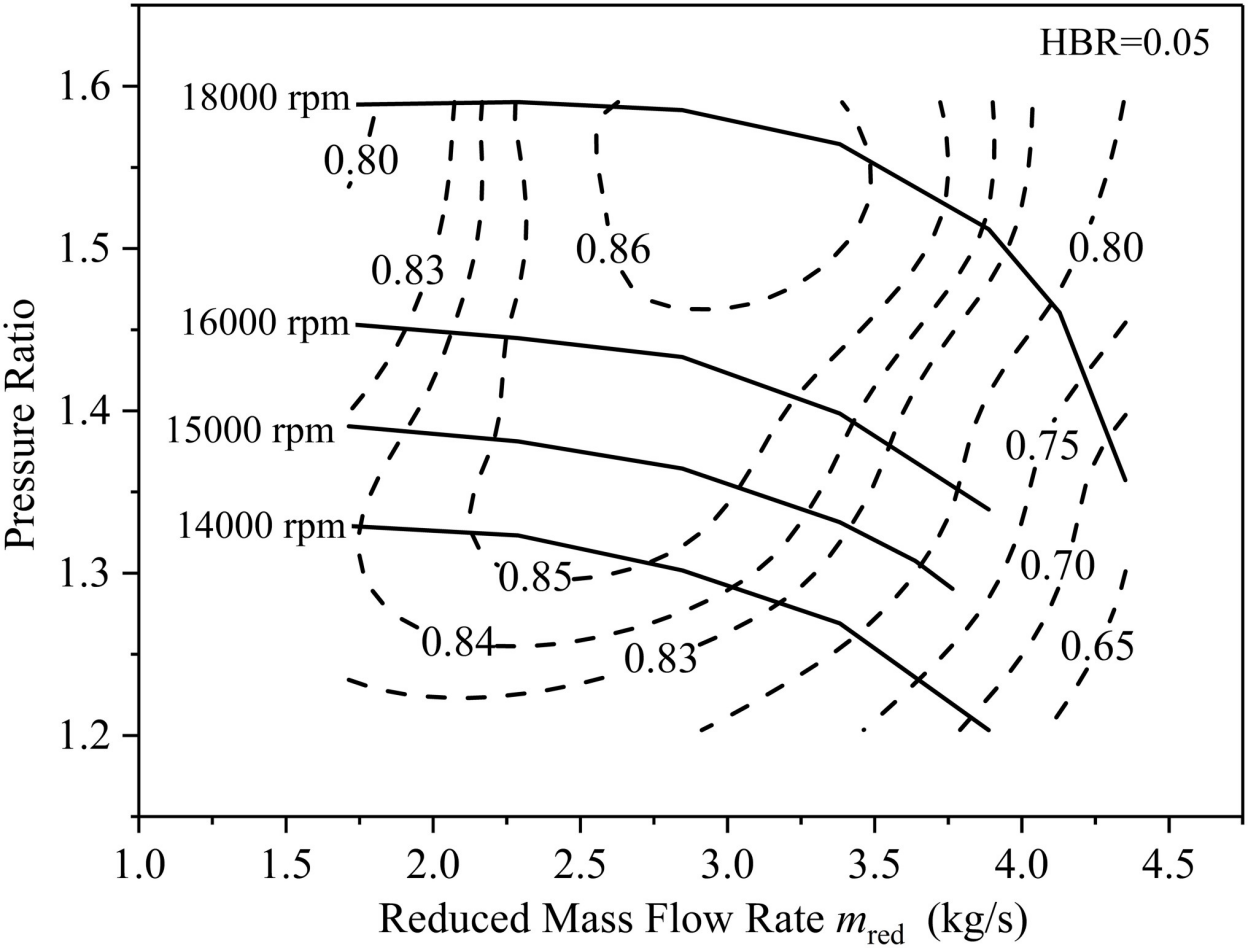
The performance map of the compressor when the HBR is 0.05.

Fig 8 is the performance map of the compressor when the HBR is 0.05. The dotted line is an equal isentropic efficiency curve, which can clearly show the distribution of isentropic efficiency in the mass flow-pressure ratio diagram. It can be found that when the flow rate is constant, the pressure ratio of the compressor increases with the increase of the rotational speed. In order to solve the problem of reducing the efficiency and pressure ratio of the compressor after hydrogen blending, increasing the speed is an effective measure. However, the speed cannot exceed a certain range. Because the high-speed rotating impeller will increase the sound speed inside the gas, which will cause the impeller to bear excessive stress. Therefore, the working point of the compressor should be reasonably determined according to the performance map corresponding to different hydrogen blending ratios.

**Fig 9:**
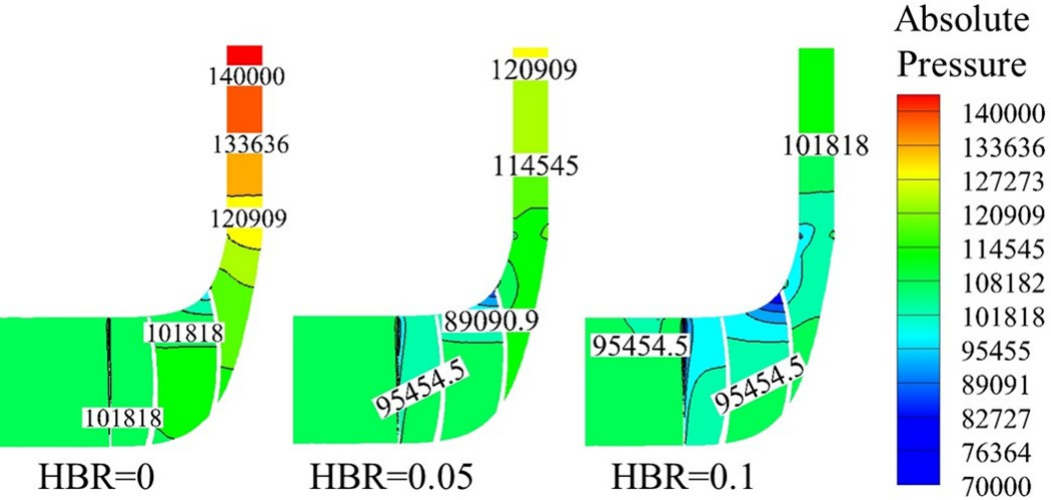
The local pressure distribution of the section *X* = 0.

In order to study the flow mechanism of the impeller, Fig 9 shows the local pressure distribution of the section *X* = 0 corresponding to different HBRs when the flow rate is 3.53 kg/s. It can be observed that the pressure is gradually increasing from the impeller inlet to the diffuser outlet. With the increase of HBR, the outlet pressure of the diffuser gradually decreases, which further indicates that hydrogen-mixed natural gas will reduce the compression efficiency of the compressor.

### 3.2. Inlet temperature

**Fig 10:**
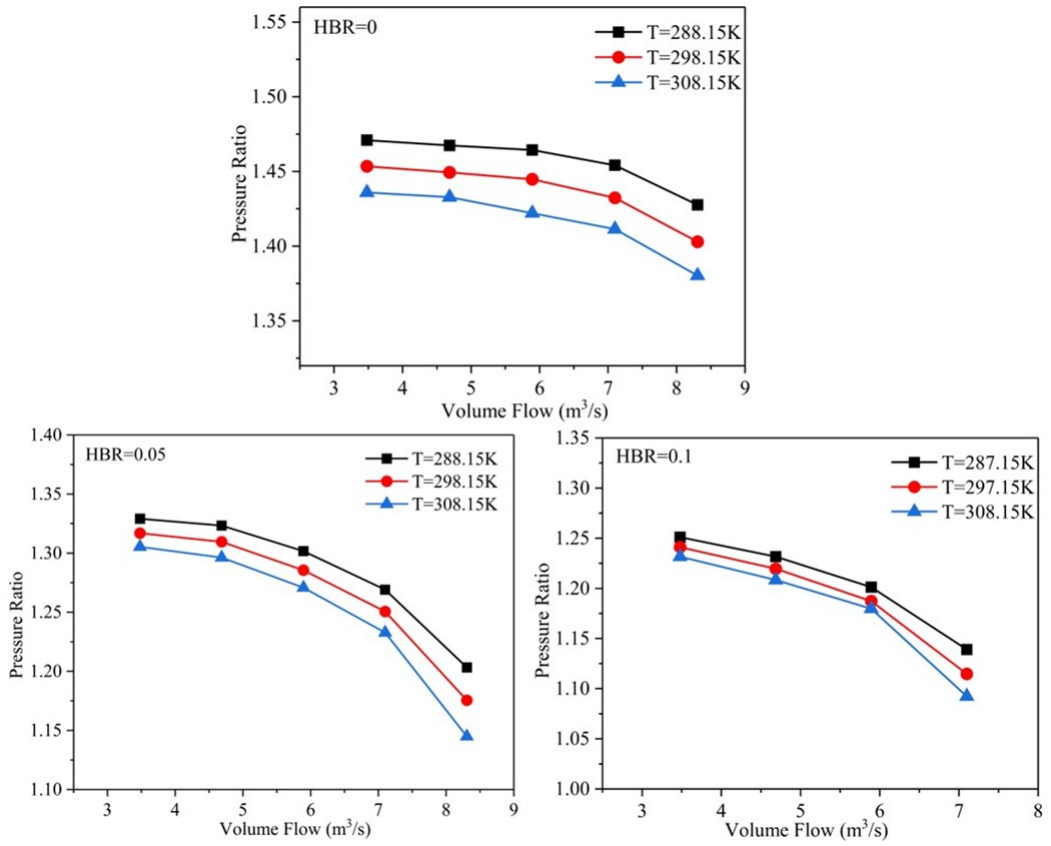
The trends of volume flow rate and pressure ratio at different inlet temperatures.

The inlet temperature of hydrogen-mixed natural gas changes with the change of environment temperature. In order to study the influence of different inlet temperatures on the aerodynamic performance of the compressor, the inlet temperatures are assumed to be 288.15 K, 298.15 K and 308.15 K, respectively. Fig 10 shows the trends of volume flow rate and pressure ratio at different inlet temperatures when the HBR is 0, 0.05 and 0.1. It can be found that the pressure ratio decreases with the increase of inlet temperature at the same volume flow rate, regardless of the hydrogen doping ratio. The rate of pressure ratio reduction is higher than that of hydrogen doping ratio of 0.05 and 0.01, when the hydrogen doping ratio is 0. This is because when the hydrogen blending ratio is 0, the working medium is pure natural gas, and its physical parameters have a significant effect on the pressure ratio.

**Fig 11:**
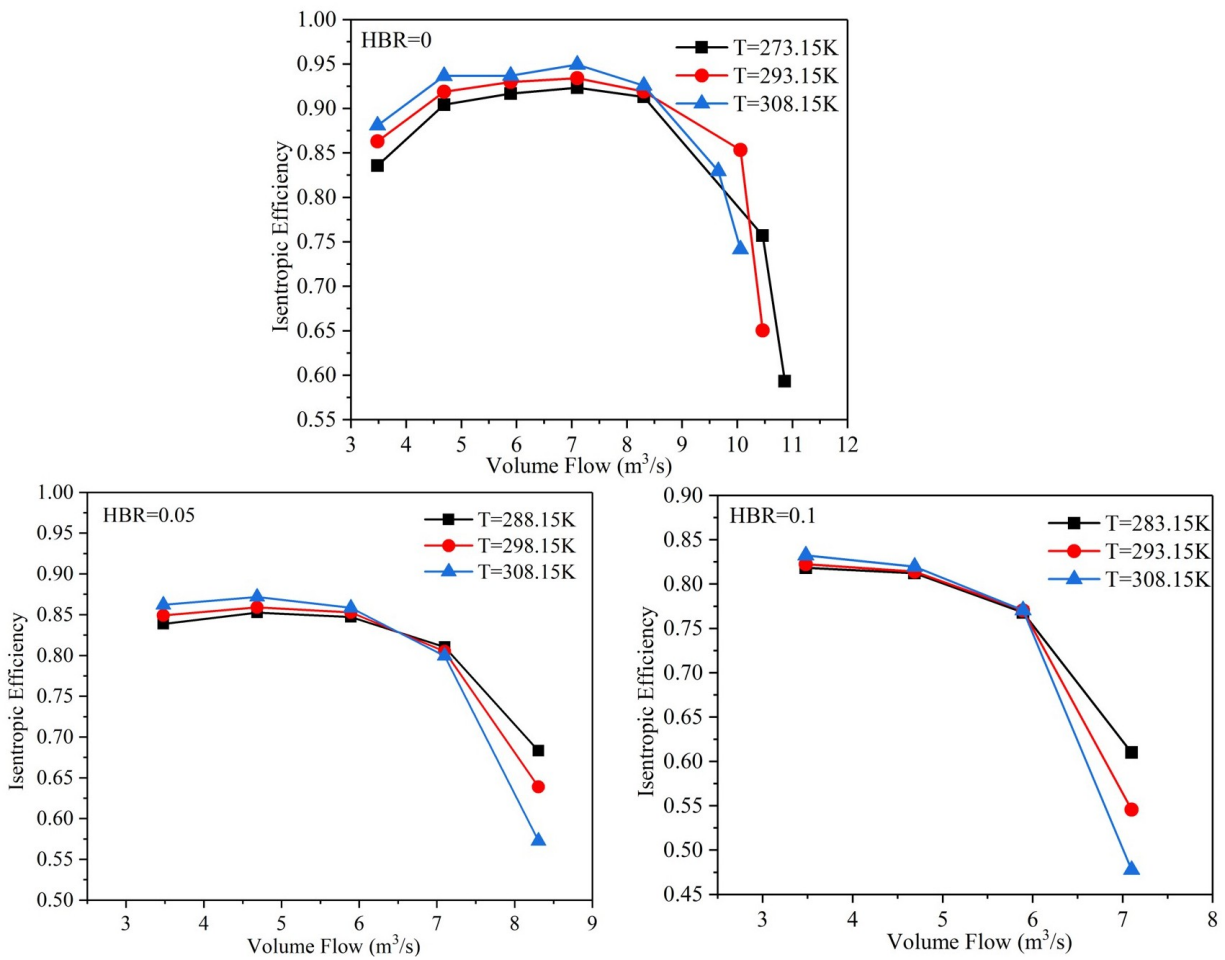
The changes of volume flow rate and isentropic efficiency at different inlet temperatures.

The changes of compressor flow and isentropic efficiency at different inlet temperatures are shown in Fig 11. It can be found that when the flow increases to a certain value, the isentropic efficiency decreases rapidly regardless of the HBR. When the inlet temperature is higher, the corresponding isentropic efficiency decreases faster. The main factors affecting the isentropic efficiency include pressure ratio and temperature difference between inlet and outlet. Under the combined effect of these two factors, the isentropic efficiency shows a rapid decrease. Therefore, high inlet temperature can improve the isentropic efficiency in the early stage of flow increase. However, in the later period, the phenomenon of sudden drop in efficiency is very easy to occur.

The process of impeller consuming external work to compress gas pressurization can be regarded as a stable flow. This process is considered as isentropic compression. According to the first law of thermodynamics, the specific aerodynamic work of compressor is defined as:

$$w_{s}=-\Delta h=\frac{\kappa }{\kappa -1}R_{g}T_{1}\left[1-{\left(\frac{p_{2}}{p_{1}}\right)}^{\left(\kappa -1\right)/\kappa }\right]$$ (10)

where *R*_g_ is the gas constant, which is the ratio of the general gas constant to the molar mass.

**Fig 12:**
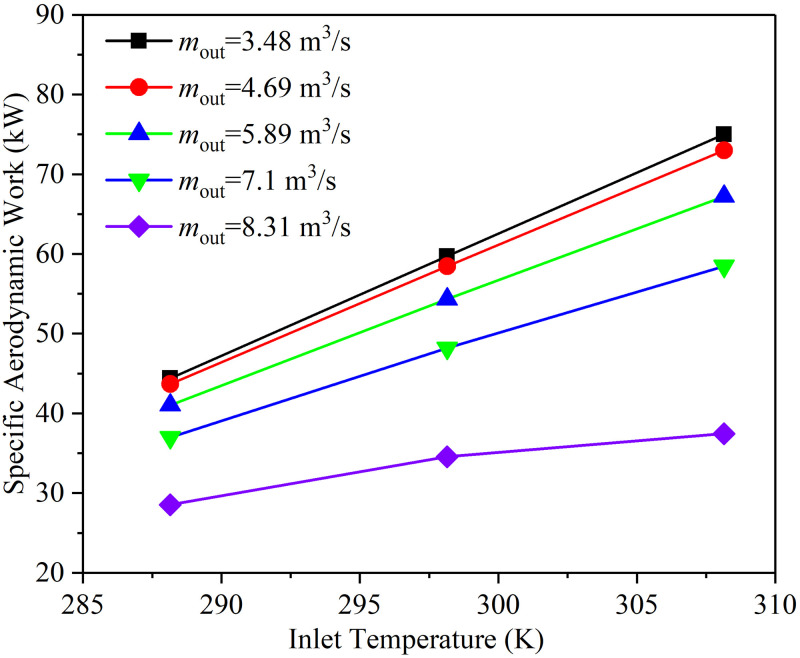
The variation of the specific aerodynamic work with the inlet temperature.

Fig 12 shows the variation of the specific aerodynamic work with the inlet temperature when the rotational speed is 14000 rpm and the HBR is 0.05. It is found that the specific aerodynamic work of the compressor increases with the increase of the inlet temperature. That is the inlet temperature of the compressor is proportional to specific aerodynamic work. This underlines the importance of inlet cooling in compressors, as the lower inlet temperature reduces the specific work required to produce the same pressure ratio.

## 4. Conclusion

Aerodynamic performance of centrifugal compressors for hydrogen-mixed natural gas is carried out by numerical model. The effects of hydrogen blend ratio and inlet temperature on compressor performance are studied. It is found that the pressure ratio of the compressor decreases with increasing hydrogen blend ratio and the isentropic adiabatic efficiency of the compressor decreases significantly at the same rotation speed. Therefore, the working range of hydrogen-mixed natural gas compressor should be reasonably designed according to hydrogen blend ratio, which is very important for the stable operation of the compressor. When the rotational speed and hydrogen blend ratio are constant, the pressure ratio decreases by no more than 4.8% as the inlet temperature increases. Therefore, the inlet temperature as a factor affecting the performance of hydrogen-doped natural gas compressors should not be ignored.

The limitation of the current research is that the influence of diffuser, volute and other components as an important part of the compressor on the performance of the compressor is not considered. In the future, the key factors affecting compressor performance can be studied for compressor models, not limited to factors such as HBR and inlet temperature. In fact, it is a good research method for centrifugal compressor model to verify the simulation results on the basis of experiments. It is necessary to carry out experimental research on centrifugal compressor.

## Supporting information

S1 Data (ZIP)
